# ZnO Quantum Dots Induced Oxidative Stress and Apoptosis in HeLa and HEK-293T Cell Lines

**DOI:** 10.3389/fphar.2020.00131

**Published:** 2020-02-27

**Authors:** Yanjie Yang, Zhenhua Song, Weixia Wu, Ao Xu, Shuangyu Lv, Shaoping Ji

**Affiliations:** Provincial Engineering Centre for Tumor Molecular Medicine, School of Basic Medical Sciences, Henan University, Kaifeng, China

**Keywords:** ZnO quantum dots, cytotoxicity, reactive oxygen species, mitochondria membrane potential, apoptosis

## Abstract

Zinc oxide (ZnO) quantum dot (QD) is a promising inexpensive inorganic nanomaterials, of which potential toxic effects on biological systems and human health should be evaluated before biomedical application. In this study, the cytotoxicity of ZnO QDs was assessed using HeLa cervical cancer cell and HEK-293T human embryonic kidney cell lines. Cell viability was significantly decreased by treatment with 50 µg/ml ZnO QDs after only 6 h, and the cytotoxicity of ZnO QDs was higher in HEK-293T than in HeLa cells. ZnO QDs increased the level of reactive oxygen species and decreased the mitochondria membrane potential in a dose-dependent manner. Several gene expression involved in apoptosis was regulated by ZnO QDs, including bcl-2 gene and caspase. In HeLa cells, ZnO QDs significantly increased early and late apoptosis, but only late apoptosis was affected in HEK-293T cells. These findings will be helpful for future research and application of ZnO QDs in biomedicine.

## Introduction

Zinc oxide nanoparticles (ZnO NPs) are one of the most extensively used nanomaterials, due to the unique physicochemical properties and low cost. ZnO NPs could protect the cells against the ultraviolet (UV)-induced skin damage, and have been applied to cosmetics (Vallabani et al., 2019). ZnO NPs are used in the packaging and food industry as additives owing to their antimicrobial properties (Sharma et al., 2012; Kim et al., 2019). ZnO NPs are also being explored for biomedical applications, including pharmaceuticals, dental fillings, drug delivery, biosensors, and bioimaging (Kim et al., 2019; Vallabani et al., 2019). With the expanded production and application of ZnO NPs, the concerns of their potential toxicity in human health and environmental safety have raised (Song et al., 2019). ZnO NPs were reported to induce cytotoxicity in a variety of cancer cells, including HepG2, U87, MCF-7 etc. (Roshini et al., 2017). Moreover they can accumulate in various internal organs (such as liver, spleen, lungs, kidney, and heart) *via* circulation and then produce adverse consequences (Shen et al., 2019).

Quantum dots (QDs) with a diameter of 1–10 nm, have unique optical properties, such as broad excitation, narrow emission spectra, and long fluorescence lifetimes (Yaghini et al., 2018; Lu et al., 2019; Touaylia and Labiadh, 2019). These advantages make QDs attractive for biomedical applications, including fluorescent probes, clinical diagnostic, and therapeutic tools (Yaghini et al., 2018). Commercially available cadmium-based QDs release toxic cadmium ions, which limit their future applicability, particularly in view of environmental regulations (Sudhagar et al., 2011; Aboulaich et al., 2012). ZnO QDs are promising and have received much attention because of their Cd-free, inexpensive, and excellent optical properties (Roshini et al., 2017). The widespread application inevitably cause the increased release into the environment. Therefore, it is necessary to evaluate the potential toxic effects of ZnO QDs.

Our previous studies have demonstrated that ZnO QDs are located in the mitochondria, disrupt the activity of anti-oxidative enzymes, and induce oxidative stress after intravenous injection in mice (Yang et al., 2014b). In this study, we assessed the cytotoxicity of ZnO QDs in HeLa cervical cancer cell and HEK-293T human embryonic kidney cell lines, and investigated the potential effect on reactive oxygen species (ROS) production, loss of mitochondria membrane potential (MMP), and promotion of apoptosis.

## Materials and Methods

### Materials

Fetal bovine serum was purchased from Hyclone (Logan, UT, USA). Dimethyl sulfoxide was purchased from Sigma–Aldrich (St Louis, MO, USA). Dulbecco's modified Eagle's medium (DMEM), phosphate-buffered saline (PBS), and trypsin were purchased from Corning (New York, NY, USA). TRIzol reagent was purchased from Invitrogen (Carlsbad, CA, USA). Cell Counting Kit-8 (CCK-8), ROS color reagent 2′,7′-dichlorodihydrofluorescein diacetate (DCFH-DA), and 5,50,6,60-tetrachloro-1,10,3,30-tetraethylbenzimidazolcarbocyanine iodide (JC-1) were purchased from Beyotime (Shanghai, China). Deionized water was prepared from Millipore (Bedford, MA, USA). Other reagents used were of analytical grade.

The synthesis of ZnO QDs were as described previously (Yang et al., 2014a). The reaction was carried out at room temperature by consecutive dropwise addition of 450 ml 0.2 mol/L KOH in ethanol to 150 ml 0.1 mol/L ZnAc_2_ in ethanol with stirring, and stirred for another 1 h. The obtained ZnO QDs was centrifuged and washed with absolute ethanol three times, then dried in vacuum. The characterization of ZnO QDs was described in **Supplemental Material**.

### Cell Culture

HeLa and HEK-293T cells were cultured in DMEM containing 10% fetal bovine serum and 1% penicillin–streptomycin. The cell lines were maintained at 37°C in a humidity- and CO_2_-controllable incubator (Thermo Forma, Marietta, OH, USA) with 5% CO_2_. All the cell experiments were performed in a clean atmosphere.

### Cell Viability Assay

Cell viability was determined by the CCK-8 assay. HeLa and HEK-293T cells were seeded into 96-well plates (2.0×10^3^ cells per well), and incubated at 37°C for 24 h. ZnO QDs in DMEM were added at final concentrations of 25, 50, 100, 200, and 400 µg/ml. At the end of the fixed incubation period (6, 24, 48, and 72 h), cells were washed with PBS to remove excess QDs. We added 200 µl fresh medium and 20 µl CCK-8 reagent. After 2 h incubation at 37°C, absorbance at 450 nm was recorded using a microplate reader. All experiments were performed in triplicate.

### Measurement of Reactive Oxygen Species Production

ROS were determined using DCFH-DA. A total of 5.0×10^4^ HeLa or HEK-293T cells per well were cultured with 0, 50, and 100 µg/ml ZnO QDs for 1, 2, 4, 6, and 24 h. Then added 10 µM DCFH-DA working solution, and the cells were incubated for 20 min at 37°C. The plates were rinsed with serum-free medium three times to remove DCFH-DA. Fluorescence intensity was detected using a microplate counter at an excitation wavelength of 488 nm and emission wavelength of 525 nm.

### Measurement of MMP (ΔѰm)

Changes in MMP were detected using a mitochondrion-specific cationic dye JC-1. HeLa and HEK-293T cells were incubated with 0, 50, and 100 µg/ml ZnO QDs for 24 h. We added JC-1 working solution and incubated the cells at 37°C in the dark for 20 min. We rinsed the cells with JC-1-free working solution three times, and replaced it with serum-free medium. Fluorescence intensity was recorded using a microplate counter. Red emission were at excitation an wavelength of 525 nm and emission wavelength of 590 nm, indicating membrane-potential-dependent JC-1 aggregates in mitochondria, while green fluorescence was detected at excitation wavelength of 490 nm and emission wavelength 530 nm, indicating the monomeric form of JC-1 in the cytoplasm. Mitochondrial depolarization resulted in a decrease in the red/green fluorescence intensity ratio.

### Total RNA Isolation and Quantitative Real-Time PCR Analysis of Apoptotic Markers

After incubation with control and 50 µg/ml ZnO QDs for 6 and 24 h, cells were collected. Total RNA was extracted using the TRIzol method and quantified by NanoDrop 2000 UV-Vis Spectrophotometer (Thermo Scientific, Wilmington, DE, USA). Total RNA (1 μg) was reverse transcribed to synthesize complementary DNA (cDNA) using the High Capacity cDNA Reverse Transcription kit (Applied Biosystems, Foster City, CA, USA). Then, the messenger RNA (mRNA) expression levels of apoptotic markers including *Bax*, *Bak*, *Bcl2*, *Bcl-xl*, *Casp3*, *Casp7*, *Casp9*, and *p53,* were assessed using quantitative real-time PCR. Real-time PCR was performed by the 7500HT Thermal Cycler and SYBR Green Master Mix (Applied Biosystems). Primers were designed following previous reports (Bai et al., 2015; Czemplik et al., 2016; Sun et al., 2016), and the primers were as follows: *Bax* forward primer: 5′-AGGGTTTCATCCAGGATCGAGCAG-3′ and reverse primer: 5′-ATCTTCTTCCAGATGGTGAGCGAG-3′, *Bak* forward primer: 5′-GGATTGGTGGGTCTATGTTC-3′ and reverse primer: 5′-TCTGGGATTCCTAGTGGTGT-3′, *Bcl2* forward primer: 5′-GCTGAGGCAGAAGGGTTATG-3′ and reverse primer: 5′-GCCCCCTTGAAAAAGTTCAT-3′, *Bcl-xl* forward primer: 5′-GATCCCCATGGCAGCAGTAAAGCAAG-3′ and reverse primer: 5′-CCCCATCCCGGAAGAGTTCATTCACT-3′, *Capse3* forward primer: 5′-AGCCCATTTCTCCATACG-3′ and reverse primer: 5′-TTATTGCCTCACCACCTTTAG-3′, *Caspase7* forward primer: 5′-CATGCGATCCATCAAGACCA-3′ and reverse primer: 5′-GGAAGCACTTGAAGAGCG-3′, *Caspase9* forward primer: 5′-TTGTCGAAGCCAACCCTA-3′ and reverse primer: 5′-GCCAAATCTGCATTTCCC-3′, *p53* forward primer: 5′-ACATGACGGAGGTTGTGA-3′ and reverse primer: 5′-CACCACCACACTATGTCG-3′, *β-actin* forward primer: 5′-GGCGGACTATGACTTAGTTG-3′ and reverse primer: 5′-AAACAACAATGTGCAATCAA-3′. All samples were analyzed in duplicate measuring both the gene of interest and internal control of *β-actin*. Dissociation curve analysis was performed after each quantitative real-time PCR. Each experiment contained three technical repetitions, and each gene was independently detected three times. The normalized expression of the target genes was calculated using 2^−ΔΔCT^.

### Apoptosis Measurement by Flow Cytometry

An Annexin V Apoptosis Detection Kit (Multisciences, Hangzhou, China) was utilized to measure apoptosis of HeLa and HEK-293T cells. After incubation with control and 50 µg/ml ZnO QDs for 24 h, cells were washed, trypsinized, and resuspended in the binding buffer at a concentration of 10^6^ cells/ml. We added fluorescein isothiocyanate–annexin V and propidium iodide, and incubated the cells in the dark for 15 min at room temperature. Apoptotic rate was analyzed using flow cytometry.

### Statistical Analysis

All the data are presented as mean ± SEM. Multiple group comparisons of the means were evaluated by one-way analysis of variance (ANOVA) followed Dunnett's test using SPSS version 16.0. The unpaired t-test was used to test the difference between the two groups. *p* < 0.05 was considered statistically significant.

## Results

The average diameter of ZnO QDs was approximately 7.10 ± 0.30 nm according to transmission electron microscopy (TEM, **Figure S1**) and 7.43 nm from dynamic light scattering (DLS, **Figure S2A**). Zeta potential was −3.56 mV on the surface of ZnO QDs (**Figure S2B**). The UV-Vis absorption was located at 363 nm (**Figure S2C**) and their strong fluorescence centered at 552 nm (**Figure S2D**).

### Cytotoxicity of ZnO Quantum Dots in HeLa and HEK-293T Cells

**Figure 1:**
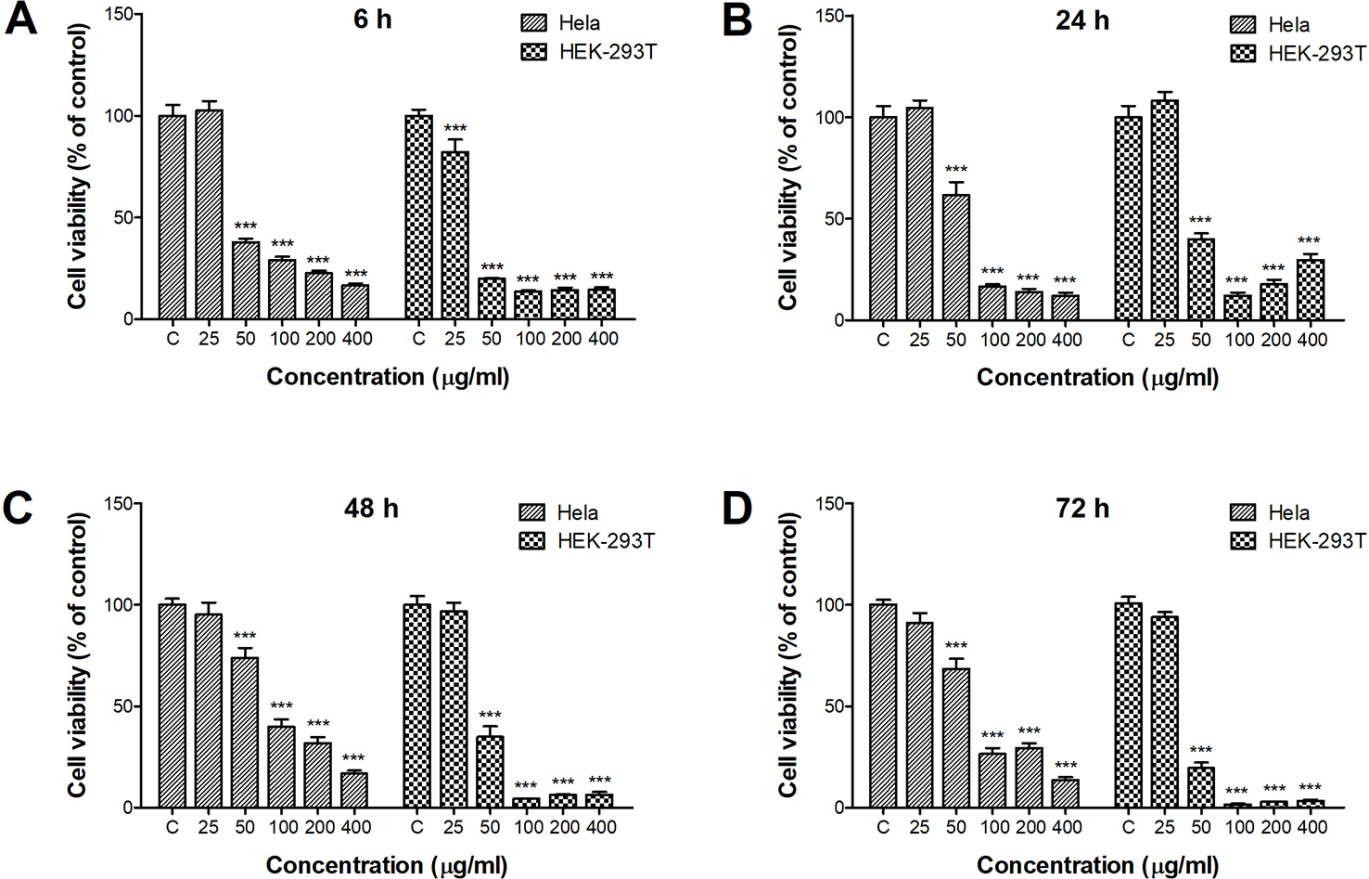
Cytotoxicity of ZnO quantum dots (QDs) against HeLa and HEK-293T cells after **(A)** 6 h, **(B)** 24 h, **(C)** 48 h, and **(D)** 72 h exposure. Cell viability determined by the CCK-8 assay was calculated relative to negative controls. All data are presented as mean ± SEM (n = 3). ***p < 0.001 *versus* control according to ANOVA followed by Dunnett's test.

The CCK-8 assay was used to examine the viability of HeLa and HEK-293T cells after incubation with ZnO QDs (**Figure 1**). Obvious cytotoxicity was observed after only 6 h incubation with ZnO QDs (**Figure 1A**). Cell viability was decreased when the concentration of QDs increased. Significant cytotoxicity appeared at a concentration of 50 µg/ml in HeLa and HEK-293T cells, and the dosage was selected for following assays. The cytotoxicity of ZnO QDs in HEK-293T cells was greater than in HeLa cells. Incubation time did not significantly influence the viability of HeLa cells, while the viability of HEK-293T cells displayed a time-dependent decrease (**Figures 1B–D**).

### Reactive Oxygen Species Induced by ZnO Quantum Dots

**Figure 2:**
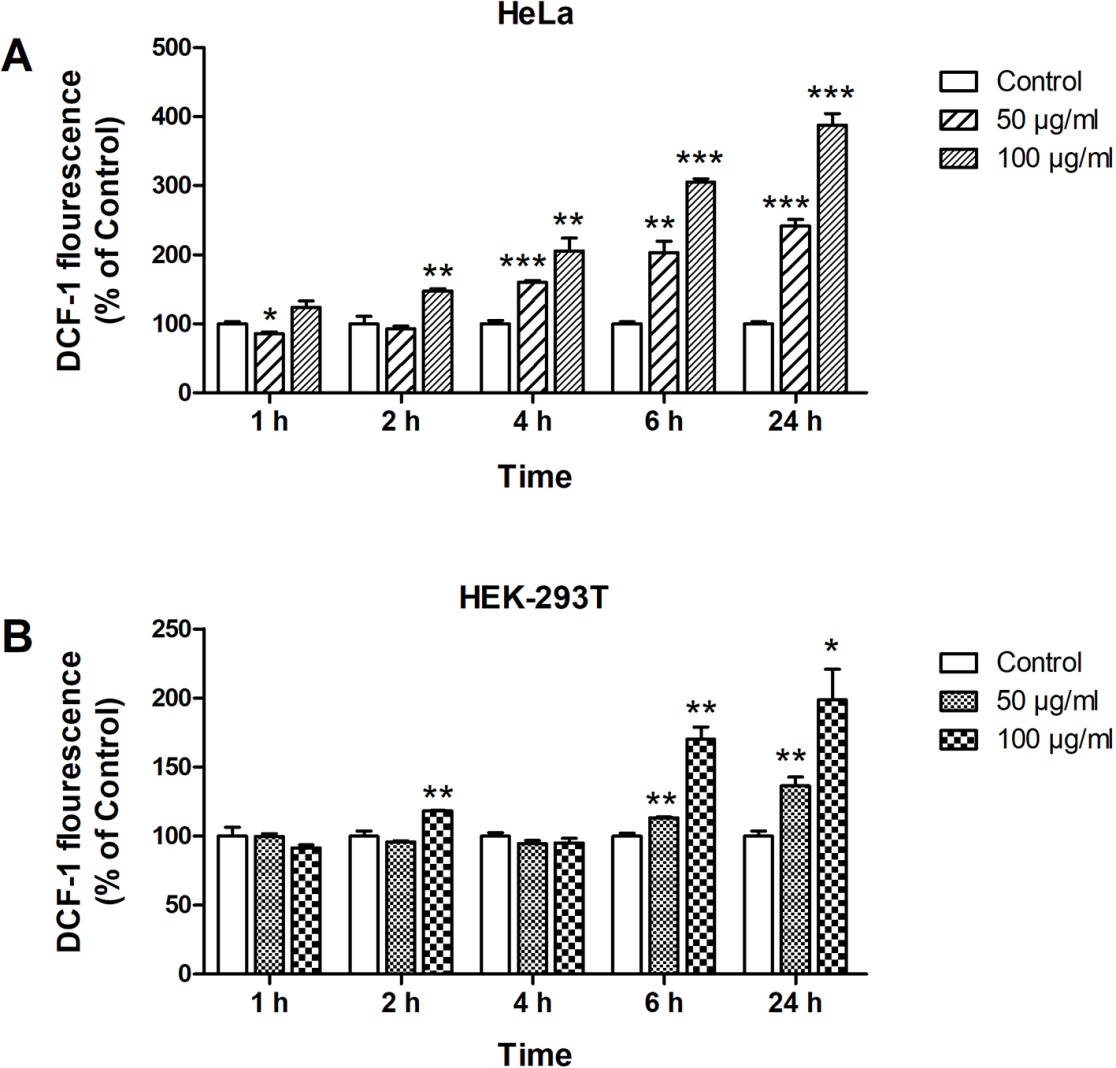
Formation of reactive oxygen species (ROS) in HeLa **(A)** and HEK-293T **(B)** cells after 1, 2, 4, 6, and 24 h incubation with 50 and 100 µg/ml of ZnO quantum dots (QDs). Dichlorofluorescein (DCF) fluorescence intensity was calculated relative to negative controls. All data are presented as mean ± SEM (n = 3). *p < 0.05, **p < 0.01 and ***p < 0.001 *versus* control according to ANOVA followed by Dunnett's test.

ROS generation by ZnO QDs was determined by DCF fluorescence. The fluorescence intensity were the same as control group until 4 h after ZnO QDs treated in HEK-293T cells (**Figure 2B**), which were significantly increased at 4 h in HeLa cell (**Figure 2A**). The fluorescence intensity induced by ZnO QDs at 100 µg/ml was stronger than at 50 µg/ml (dose dependent), and highest at 24 h (time dependent) in HeLa and HEK-293T cells. The fluorescence intensity in HeLa cells was stronger than in HEK-293T cells at the same incubation time and dose.

### Loss of MMP Induced by ZnO Quantum Dots

**Figure 3:**
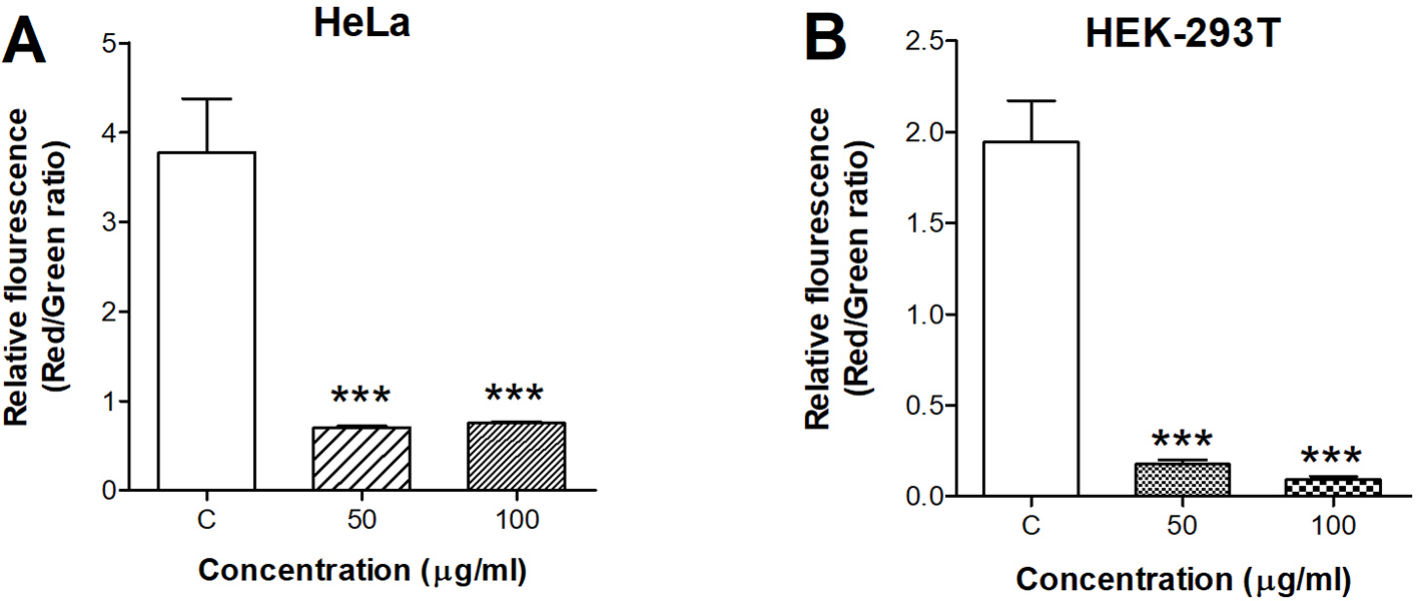
Quantitative analysis of membrane potential after incubation with 50 and 100 µg/ml ZnO quantum dots (QDs) for 24 h. **(A)** HeLa and **(B)** HEK-293T cells. All data are presented as mean ± SEM (n = 3). ***p < 0.001 *versus* control according to ANOVA followed by Dunnett's test.

We applied the fluorescent dye JC-1 to investigate the influence of ZnO QDs on ΔΨm. When ΔΨm collapsed, JC-1 aggregates were unable to accumulate in the mitochondria and dissipated into JC-1 monomers, as a result of a decrease in the ratio of red to green fluorescence. The red/green ratio in HeLa cells was dramatically decreased from 3.77 ± 0.61 (control) to 0.70 ± 0.02 (50 µg/ml, *p* < 0.001) and 0.76 ± 0.01 (100 µg/ml, *p* < 0.001) after incubation with ZnO QDs for 24 h (**Figure 3A**). The red/green ratio was decreased from 1.94 ± 0.23 (control) to 0.17 ± 0.03 (50 µg/ml, *p* < 0.001) and 0.10 ± 0.01 (100 µg/ml, *p* < 0.001) in HEK-293T cells (**Figure 3B**).

### Quantitative Real-Time PCR

**Figure 4:**
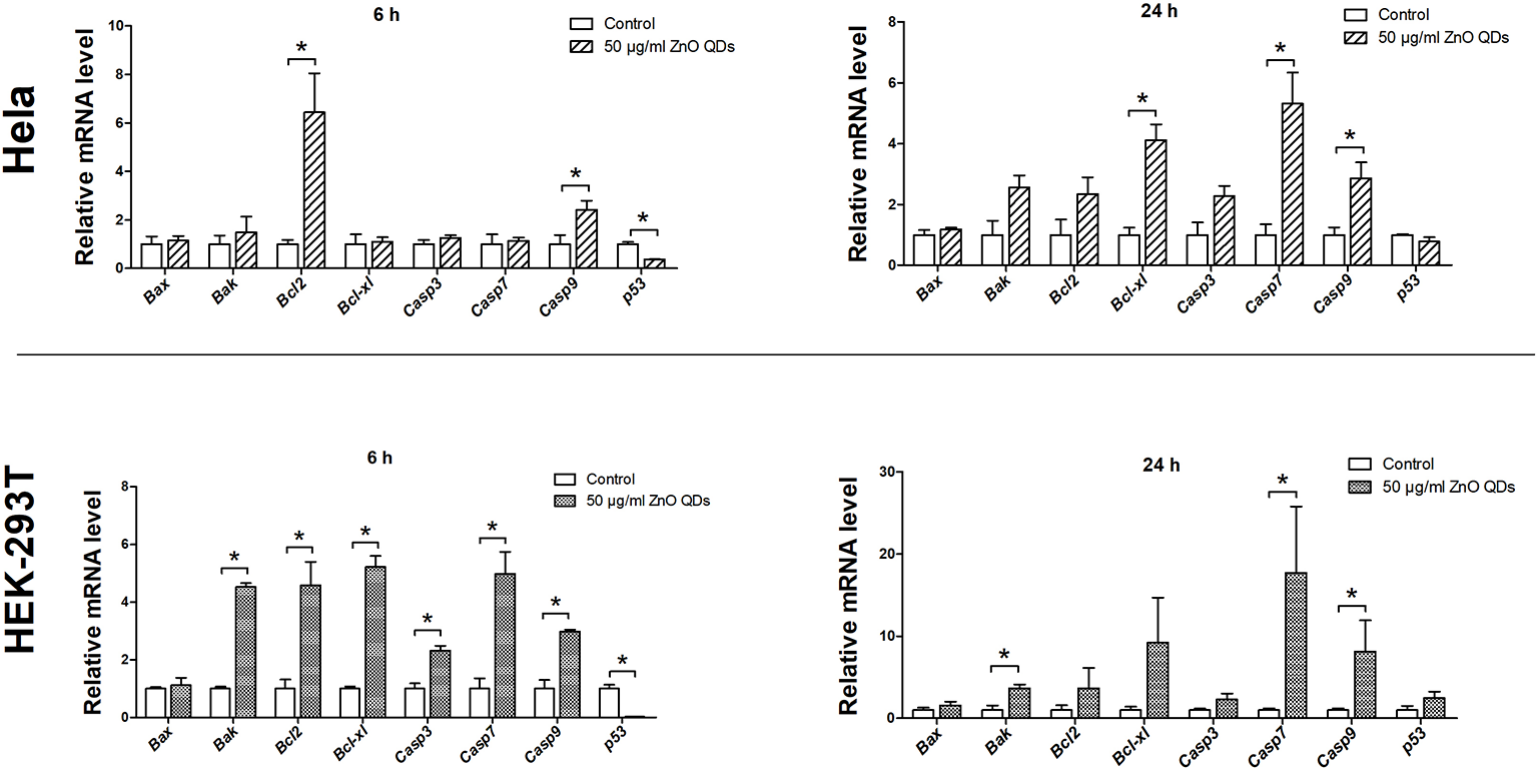
Changes in messenger RNA (mRNA) expression levels of apoptotic markers in HeLa and HEK-293T cells after exposure to 50 µg/ml ZnO quantum dots (QDs) for 6 and 24 h. All data are presented as mean ± SEM (n = 3). *p < 0.05 according to the unpaired t-test between 50 µg/ml ZnO QDs and the control group.

We used quantitative real-time PCR to analyze the mRNA levels of apoptotic markers in HeLa and HEK-293T cells after incubation with 50 µg/ml ZnO QDs for 6 and 24 h (**Figure 4**). At 6 h, only Bcl-2 (5.02-fold), caspase-9 (2.42-fold), and p53 (0.37-fold) were significantly altered in HeLa cells, while Bak (4.53-fold), Bcl-2 (4.58-fold), BCl-xl (5.22-fold), caspase-3 (2.32-fold), caspase-7 (4.98-fold), caspase-9 (2.98-fold), and p53 (0.03-fold) were all significantly altered in HEK-293T cells. mRNA levels of caspase-7 and caspase-9 were increased in HeLa and HEK-293T cells after 24 h. At the same time, mRNA expression of p53 was similar to the control groups in HeLa and HEK-293T cells.

### Apoptosis

**Figure 5:**
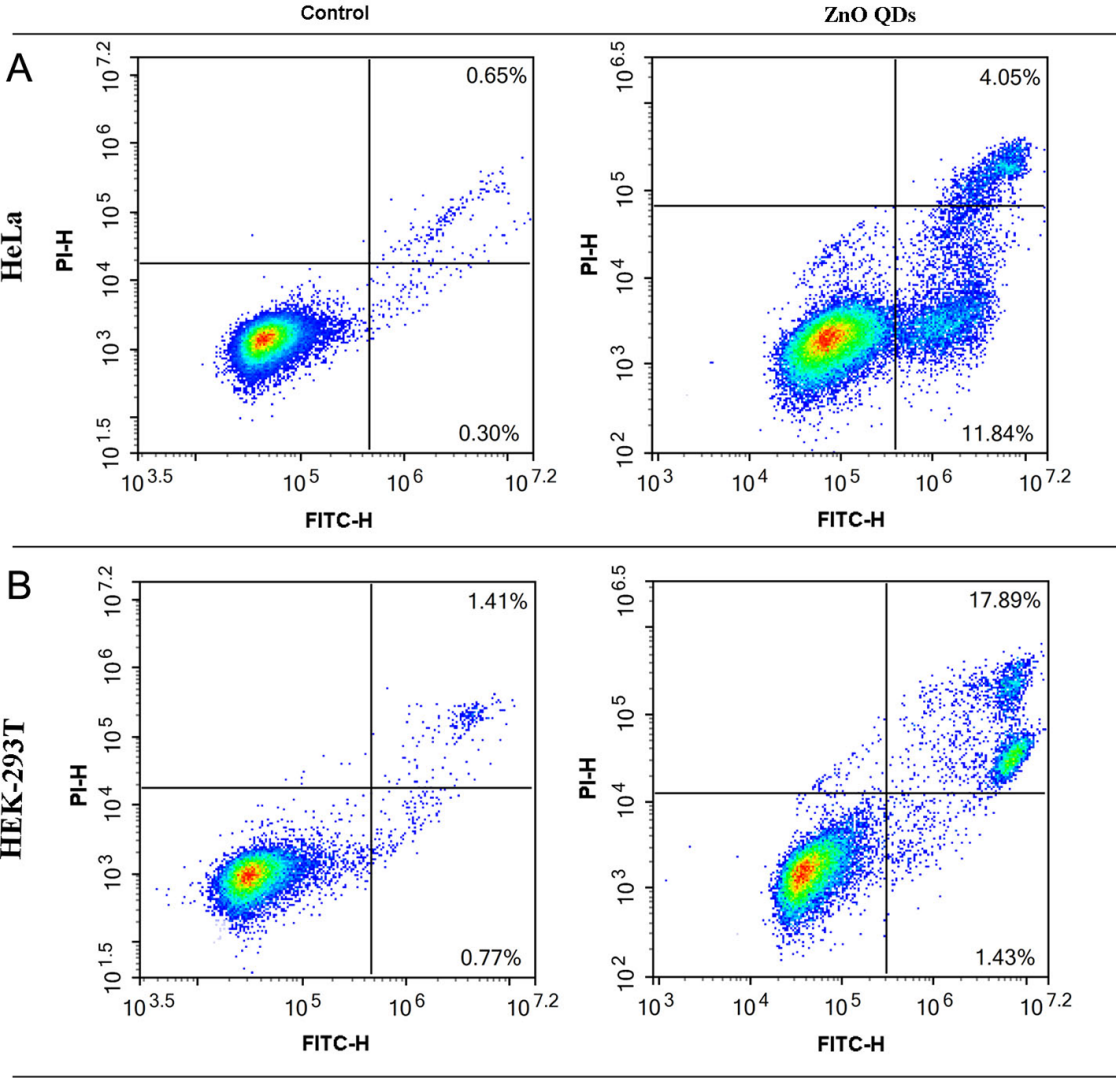
Annexin V/PI staining combined with flow cytometry was used to determine apoptosis induced by 50 µg/ml ZnO QDs treated for 24 h. **(A)** HeLa and **(B)** HEK-293T cells.

**Figure 6:**
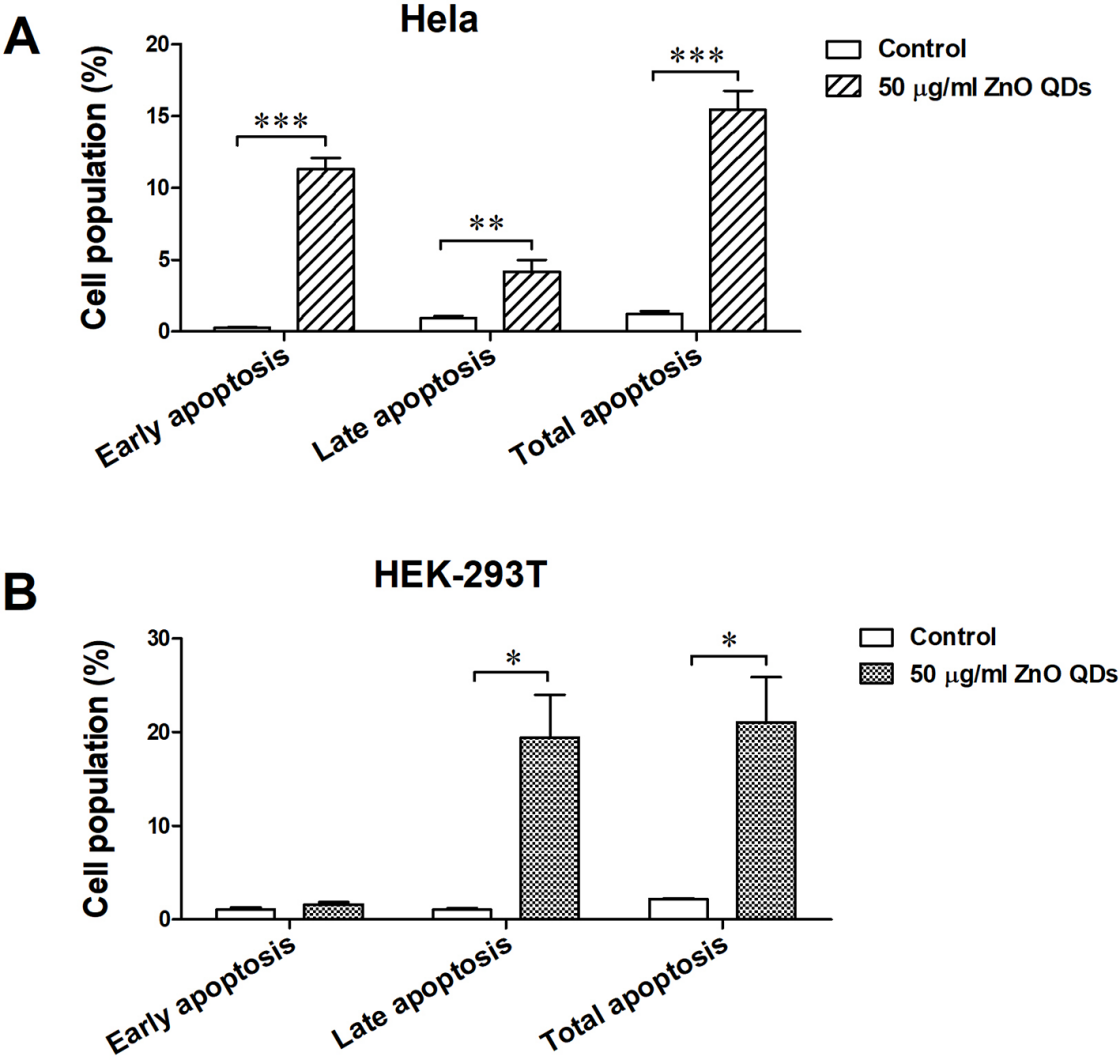
Apoptotic rate of **(A)** HeLa and **(B)** HEK-293T cells after exposure to 50 µg/ml ZnO quantum dots (QDs) for 24 h. All data are presented as mean ± SEM (n = 3). *p < 0.05, **p < 0.01 and ***p < 0.001 according to the unpaired t-test between 50 µg/ml ZnO QDs and the control group.

The percentages of apoptotic and necrotic cells in HeLa (**Figure 5A**) and HEK-293T cells line (**Figure 5B**) were analyzed by flow cytometry. Incubation with 50 µg/ml ZnO QDs for 24 h significantly increased the percentage of early apoptotic cells and late apoptotic cells in HeLa cells (**Figure 6A**). However, the cell population was located in late apoptosis in HEK-293T cells (**Figure 6B**). The total apoptosis ratio induced by ZnO QDs in HEK-293T cells (21.05 ± 4.81%) was higher than in HeLa cells (15.45 ± 1.30%). After treatment with 50 µg/ml ZnO QDs, necrotic cells remained at a low level in HeLa and HEK-293T cells.

## Discussion

In the present study, HeLa and HEK-293T cells were exposed to ZnO QDs (25, 50, 100, 200, and 400 µg/ml) for 6, 24, 48, and 72 h. The cytotoxicity was measured by CCK-8 assay. Cell viability was significantly reduced by 50 µg/ml ZnO-QDs after only 6 h treatment. This is contradicted with the result of Sudhagar et al. reports, that ZnO QDs were biocompatible and non-toxic even at 100 µg/ml for up to 48 h in MDA-MB-231 breast cancer cells (Sudhagar et al., 2011). In our study, the cytotoxicity of ZnO QDs in HEK-293T cells was greater than in HeLa cells. However, Roshini et al. reported that ZnO QDs showed less toxicity to HEK-293 cells but significantly induced cytotoxicity in human breast cancer cells (Roshini et al., 2017).

The release of ROS has been reported in cells exposed to ZnO nanoparticles, and involved in their toxic effects (Heim et al., 2015; Yang et al., 2015). Marfavi and Vallabani et al. demonstrated that ZnO NPs induced ROS in GBM U87MG and HaCaT cell line (Marfavi et al., 2019; Vallabani et al., 2019). In our study, the fluorescence intensity was increased as early as 4 h, which was consistent with the reports that ZnO NPs induces significant ROS in HaCaT cells after 3 h of treatment (Manna et al., 2015). ROS level increased in a time and dose-dependent manner, and was consistent with the result of CCK-8 assays (6 and 24 h). Therefore, we suggest that decreased cell viability was related to oxidative stress. The free radicals would lead to disorganization of the cells, organelles and enzymes (Ahmad et al., 2015). Mitochondria are especially sensitive and vulnerable to cellular stress because of their high metabolic activity (Choi et al., 2007; Manna et al., 2015). We used a specific dye JC-1 for assessment of mitochondrial damage after exposure to ZnO QDs. We detected a decrease in MMP, suggesting an impairment of mitochondrial function. This is in according with the previous reports that ZnO NPs lead to mitochondrial membrane depolarization in HaCaT cells and human bone marrow-derived MSCs (Kim et al., 2019; Vallabani et al., 2019).

Both mitochondrial membrane depolarization and free radicals could trigger apoptosis *via* the intrinsic pathway. Upon quantification by ﬂow cytometry, the total apoptosis ratio was significantly increased after exposure to 50 µg/ml ZnO QDs as compared to the control group. Persaud and Kim et al. reported that ZnO NPs induced significantly late apoptosis at the concentrations of 20 µg/ml (Kim et al., 2019; Persaud et al., 2019). Shen et al. revealed that ZnO NPs induced early and late apoptosis in low dosage, and mainly increased the late apoptosis in high dosage (Shen et al., 2019). In our experiment, ZnO QDs increased early and late apoptosis in HeLa cells, but only late apoptosis in HEK-293T cells.

## Conclusion

The cytotoxicity of ZnO QDs was evaluated in the HeLa and HEK-293T cell lines. ZnO QDs induced obvious cytotoxicity at a concentration of 50 µg/ml, and viability of HEK-293T cells was lower than that in HeLa cells. ROS level was increased in a time and dose-dependent manner by ZnO QDs and was consistent with the result of CCK-8 assays. Mitochondrial membrane depolarization and apoptosis occurred after exposure to ZnO QDs.

## Data Availability Statement

The datasets generated for this study are available on request to the corresponding authors.

## Ethics Statement

The study was approved by the Committee of Medical Ethics and Welfare for Experimental Animals, Henan University School of Medicine.

## Author Contributions

SL and SJ developed the idea and designed the research. YY, ZS, WW and AX performed the experiments. YY analyzed the data and wrote the draft of the manuscript. SJ and SL contributed to revise the writing. All authors read and approved the submitted version.

## Funding

This work was supported by research funds from the Key Science and Technology Program of Henan Province in China (Grant No. 182102310593, 192102310080), the National Natural Science Foundation of China (Grant No. 81600974, 81971280), the Key Scientific Research Program for Universities of Henan Province (Grant No. 17A330001), the Key Science and Technology Program of Kaifeng City in China (Grant No. 1903083, 1903019, 1803034), and the Research Program for Young Talent of Henan University School of Medicine (Grant No. 2019018).

## Conflict of Interest

The authors declare that the research was conducted in the absence of any commercial or financial relationships that could be construed as a potential conflict of interest.
