# Supplementary material for: ZnO Quantum Dots Induced Oxidative Stress and Apoptosis in HeLa and HEK-293T Cell Lines
Source: Front Pharmacol. 2020 Feb 27;11:131. doi: 10.3389/fphar.2020.00131 (PMC7057716; doi:10.3389/fphar.2020.00131)
Supplement: Supplementary file 1 [file DataSheet_1.doc]

**Characterization of ZnO QDs**

Transmission electron microscopy (TEM) images were measured with a JEM2100Plus (JEOL, Japan). The size distribution and zeta potentials was determined using a Nano ZS90 (Malvern, U.K.). Absorption spectra were recorded using a Shimadzu UV-1800 spectrophotometer (Japan). Fluorescence spectra were recorded on a RF-5301PC fluorescence spectrometer (Shimadzu, Japan).

The average diameter of ZnO QDs was 7.10 ± 0.30 nm (Figure S1) from TEM, which was 7.43 nm from dynamic light scattering (DLS) measurements (Figure S2A). Zeta potential was -3.56 mV on the surface of ZnO QDs (Figure S2B). The UV-Vis absorption was located at 363 nm (Figure S2C), and the emission peak was located 552 nm (Figure S2D).


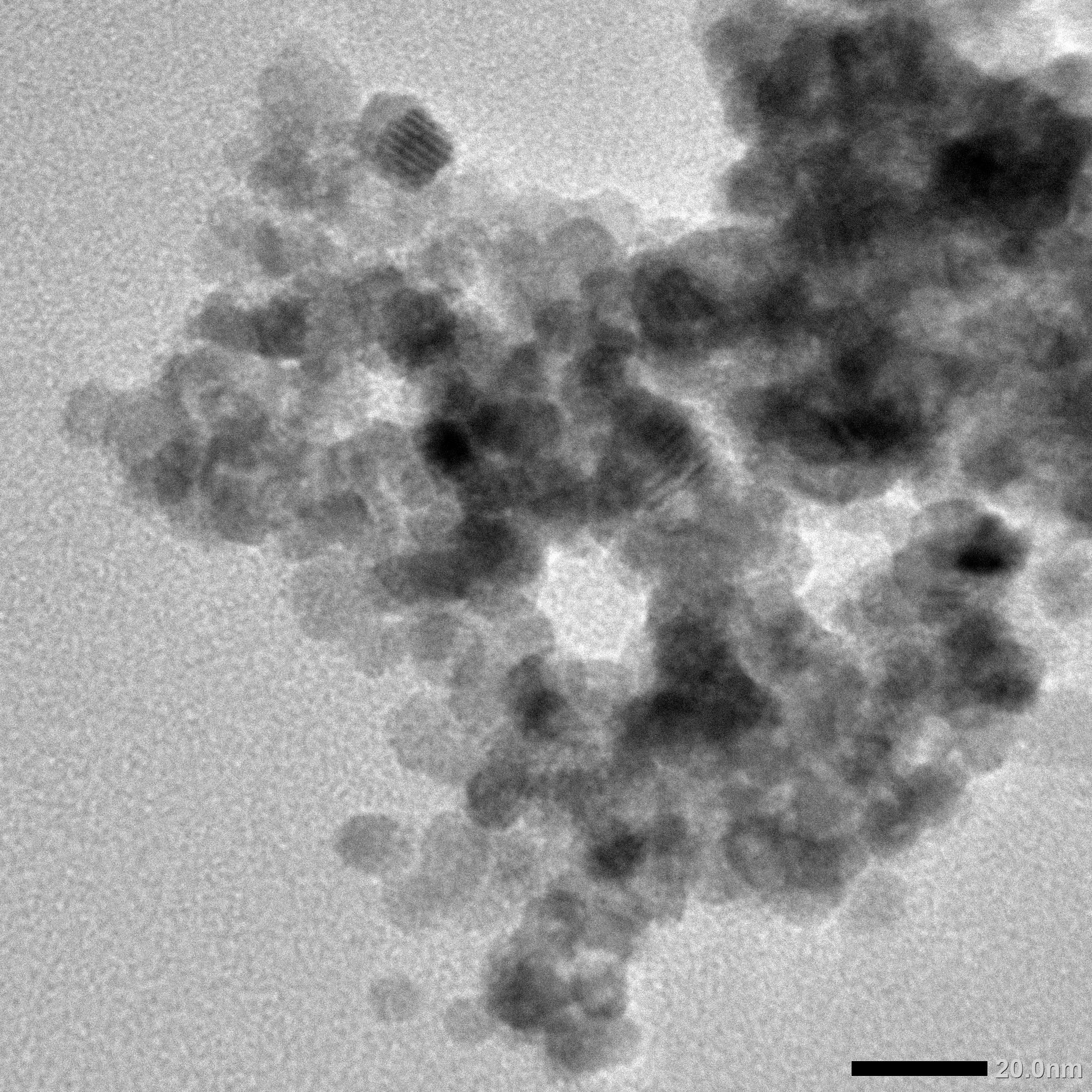


Figure S1 TEM images of ZnO QDs.


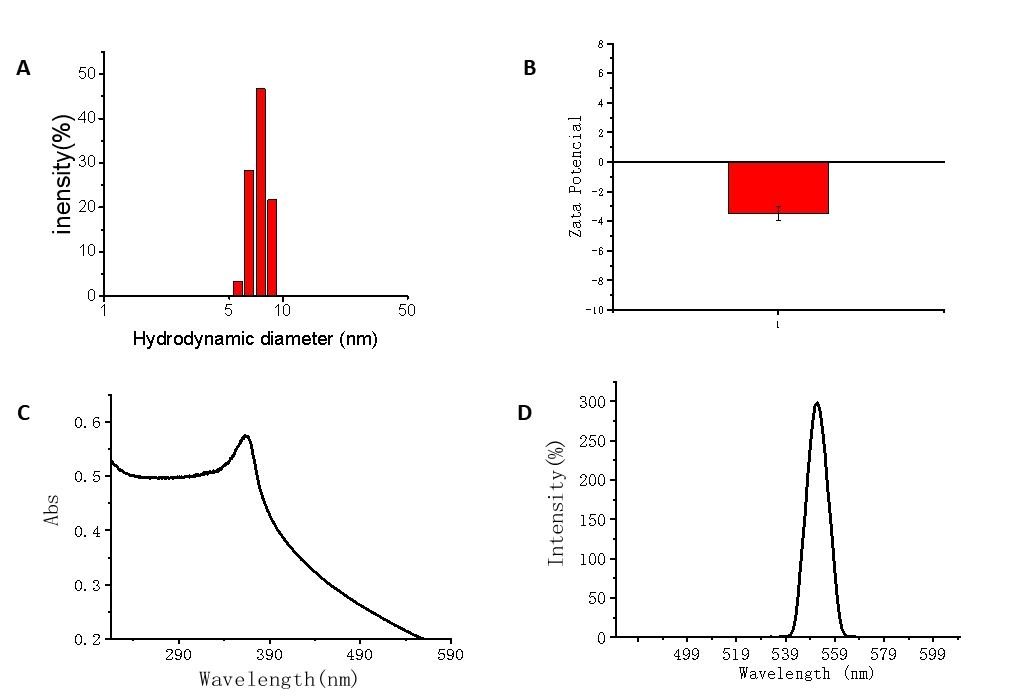


Figure S2 Characterization of ZnO QDs. (A) DLS, (B) Zeta potential, (C) UV–Vis absorption, and (D) photoluminescence (PL) spectra
